# Brain-Modulated Effects of Auricular Acupressure on the Regulation of Autonomic Function in Healthy Volunteers

**DOI:** 10.1155/2012/714391

**Published:** 2011-08-29

**Authors:** Xin-Yan Gao, Lu Wang, Ingrid Gaischek, Yvonne Michenthaler, Bing Zhu, Gerhard Litscher

**Affiliations:** ^1^TCM Research Center Graz and Research Unit of Biomedical Engineering in Anesthesia and Intensive Care Medicine, Medical University of Graz, Auenbruggerplatz 29, 8036 Graz, Austria; ^2^Department of Physiology, Institute of Acupuncture and Moxibustion, China Academy of Chinese Medical Sciences, Beijing 100700, China

## Abstract

Auricular acupuncture has been described in ancient China as well as Egypt, Greece, and Rome. At the end of the 1950s, ear acupuncture was further developed by the French physician Dr. Paul Nogier. The goal of this study was to develop a new system for ear acupressure (vibration stimulation) and to perform pilot investigations on the possible acute effects of vibration and manual ear acupressure on heart rate (HR), heart rate variability (HRV), pulse wave velocity (PWV), and the augmentation index (AIx) using new noninvasive recording methods. Investigations were performed in 14 healthy volunteers (mean age ± SD: 26.3 ± 4.3 years; 9 females, 5 males) before, during, and after acupressure vibration and manual acupressure stimulation at the “heart” auricular acupuncture point. The results showed a significant decrease in HR (*P* ≤ 0.001) and a significant increase in HRV total (*P* = 0.008) after manual ear acupressure. The PWV decreased markedly (yet insignificantly) whereas the AIx increased immediately after both methods of stimulation. The increase in the low-frequency band of HRV was mainly based on the intensification of the related mechanism of blood pressure regulation (10-s-rhythm). Further studies in Beijing using animal models and investigations in Graz using human subjects are already in progress.

## 1. Introduction

The Chinese have provided an ancient explanation of traditional acupuncture based on the principle of energy flow around the body in channels called meridians. This energy flow, called “Qi,” can be out of balance. Inserting acupuncture needles or stimulating acupoints using acupressure can reestablish harmony.

Using technology from western medicine, one can clearly measure the effects of acupuncture and acupuncture-like stimulation in the brain and periphery [1–3]. Computer-based monitoring of heart rate (HR) and heart rate variability (HRV) as well as innovative pulse wave analysis allow for diagnosis and prognosis concerning the functional state of the arteries and the heart, which is modulated by different centres of the brain [4]. Pulse wave velocity (PWV) is widely recognised as a direct marker of arterial stiffness. The augmentation index (AIx) is used more often as a parameter of wave reflection [5, 6].

Auricular acupuncture has been described in ancient China as well as Egypt, Greece, and Rome [7]. At the end of the 1950s, ear acupuncture was further developed by the French physician Dr. Paul Nogier. He systematically demonstrated that different regions of the ear and specific organs have definite functional relationships and dependencies. Due to these relationships, needling and/or stimulation of one or more ear acupuncture point can be performed to treat specific organ functions. Ear acupuncture points are also relevant for diagnostics in the field of auricular medicine. According to Nogier, a change in skin resistance at certain areas of specific ear acupuncture points is present in particular organic diseases [8–11]. 

The goal of this study was to develop a new system for acupressure (vibration stimulation) and to perform pilot investigations on possible acute effects of vibration and ear acupressure at the “heart” ear acupoint on HR, HRV, PWV and AIx in a group of healthy volunteers using new noninvasive recording methods.

## 2. Methods and Subjects

### 2.1. A New System for Ear Acupressure (Vibration Stimulation)

**Figure 1:**
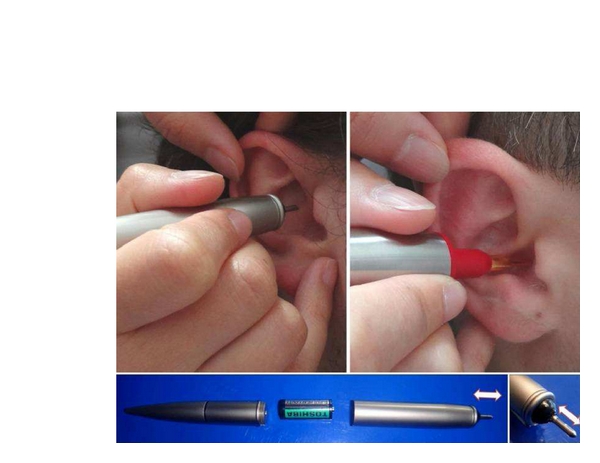
New instruments for acupressure (vibration) stimulation used in ear acupuncture at the “heart” acupoint.

Two different methods were used for stimulating the “heart” ear acupoint. The first method uses a pen with a special electronic device inside. With this pen, mechanical vibration stimuli can be administered at a frequency of about 30 Hz. The tip of the equipment is made of stainless steel (material no. 4301) and has a diameter of 2 mm and a length of 7 mm (see Figure 1, left and bottom). The vibration starts once the contact pressure reaches about 1 N (100 g bearing pressure). The second method uses a commercially available point locator (Biegler GmbH, Mauerbach, Austria) from which the battery has been removed in order to avoid acoustic stimuli (see Figure 1, right). This pen was used for manual acupressure (without vibration).

### 2.2. Recording Systems and Evaluation Parameters

An HRV medilog AR12 (Huntleigh Healthcare, Cardiff, UK, and Leupamed GmbH, Graz, Austria) system was used for electrocardiographic (ECG) monitoring. The system is designed for a monitoring period of more than 24 hours. The sampling rate of the recorder is 4096 samples per second. Therefore, R-waves can be detected accurately. All raw data are stored digitally on a 32-MB compact flash memory card. After removing the card from the portable systems, the data can be read by an appropriate card reader connected with a standard computer. The R-peak time resolution is 244 microseconds, and the *P* and *T* time resolution is 1,953 microseconds. The dimensions of the HRV recorder are 70 × 100 × 22 millimeters, and the weight is about 95 grams with batteries [12, 13].

**Figure 2:**
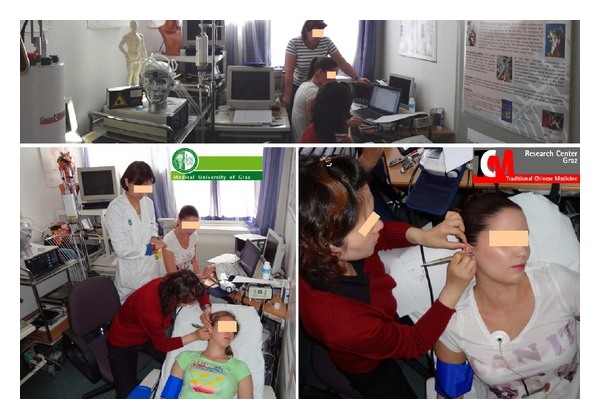
The measurements took place in the lab of the TCM Research Center in Graz at the Medical University of Graz (with permission of all medical doctors and volunteers).

HRV is measured as the percentage of change in sequential chamber complexes called RR-intervals in the ECG. The registration of HRV is performed using three electrodes (Skintact Premier F-55; Leonhard Lang GmbH, Innsbruck, Austria) on the chest (cf.  Figure 2). HRV can be quantified over time by registering the percentage changes in the RR-intervals in the time domain as well as the changes in the frequency range by analysis of the ECG power spectra. The HRV parameters are recommended by the task force of the European Society of Cardiology and the North American Society of Pacing and Electrophysiology [14]. With new software (Huntleigh Healthcare, Cardiff, UK), the HRV is analysed and displayed in a novel way to evaluate the function of the autonomic nervous system. The mean HR, the total HRV, and the LF (low frequency)/HF (high frequency) ratio of the HRV served as the evaluation parameters [14]. This innovative analysis demonstrates how well the human body reacts to acupuncture [12].

The methods for determining arterial stiffness and wave reflection parameters were noninvasive. The measurements were performed with a cuff applied to the brachial artery (cf.  Figure 2). Arteriograph (TensioMed, Budapest, Hungary) is a new, noninvasive system that uses an entirely novel method to determine PWV and AIx. Signals can be detected from an upper arm cuff, even if it is overinflated by 35–40 mmHg beyond the systolic blood pressure, despite a completely closed brachial artery. For further explanations, see [5, 6]. 

AIx describes the influence of the reflected pulse wave on systolic pressure (in percent of blood pressure amplitude) [6]. PWV describes the stiffness of the aortic vascular wall. It is considered a direct measure of (aortic) arterial stiffness [6].

### 2.3. Volunteers, Acupuncture, and Procedure

**Figure 3:**
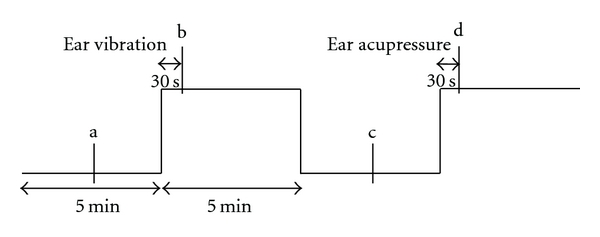
Measurement profile. Ear vibration and manual ear acupressure were performed for 30 sec each. The measurement points for wave reflection and arterial stiffness are indicated with a–d.

Within this study, 14 healthy volunteers (9 females, 5 males) with a mean age ± standard deviation (SD) of 26.3 ± 4.3 years (range 19–34 years), a mean height of 169.9 ± 6.6 cm, and a mean weight of 63.4 ± 10.7 kg were investigated. The measurement profile and measurement times (a–d) are shown schematically before, during, and after ear vibration and ear acupressure stimulation in Figure 3.

None of the volunteers were taking any medication. All volunteers were informed about the nature of the investigation as far as the study design allowed. The study was approved by the local ethics committee, and all volunteers gave their written informed consent.

The volunteers laid on a bed in our lab (see Figure 2). Room temperature was kept constant at 25°C. Four measurement periods—one before ear vibration (a), one immediately after 30 sec ear vibration (b), one in a second control section (c), and one immediately after 30 sec manual acupressure using a special instrument (d)—were compared (see Figure 3).

Acupressure stimulation was performed at the “heart” acupoint. This acupoint is one of the most important ear acupuncture points and is commonly used in patients with hypertension [15]. The “heart” auricular acupuncture point is located in the middle of the ear cavity [15–18].

### 2.4. Statistical Analysis

Data were analysed using Friedman repeated measures ANOVA on ranks (SigmaPlot 11.0, Systat Software Inc., Chicago, Ill, USA). Post hoc analysis was performed with Tukey test. The level of significance was defined as *P* < 0.05.

## 3. Results

**Figure 4:**
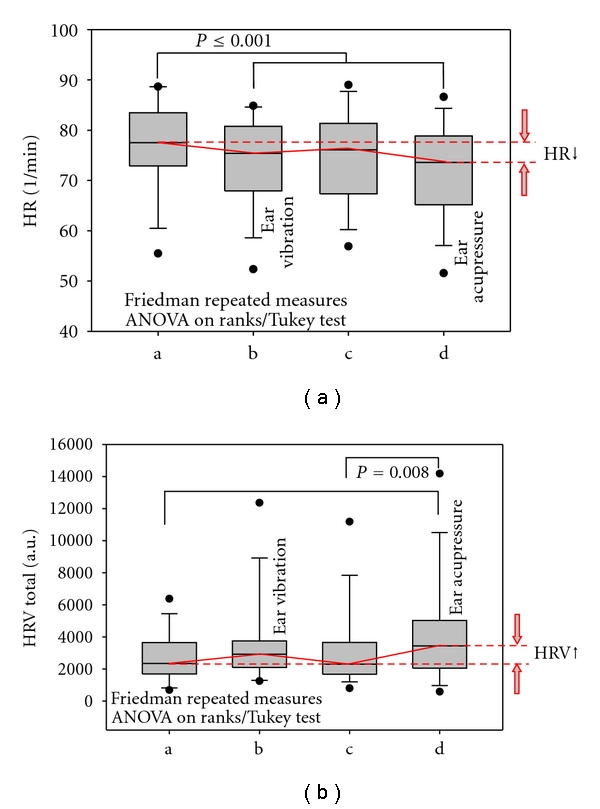
(a) mean heart rate (HR). (b) total heart rate variability (HRV total). Box plot illustrations of 14 healthy volunteers are shown. Note the significant differences. The ends of the boxes define the 25th and 75th percentiles with a line at the median and error bars defining the 10th and 90th percentiles. The different measurement phases and points (a–d) are indicated (cf Figure 3).

Figure 4 shows the mean HR and HRV total (total heart rate variability) from the ECG recordings during two control measurements (b and c) as well as during and after ear vibration at the “heart” ear acupoint (b) and manual ear acupuncture at the same point (d). There was a significant decrease of HR (*P* ≤ 0.001) during both stimulation sections. At the same time, HRV total increased significantly (*P* = 0.008) only during manual ear acupressure; however, it also increased insignificantly during ear vibration.

**Figure 5:**
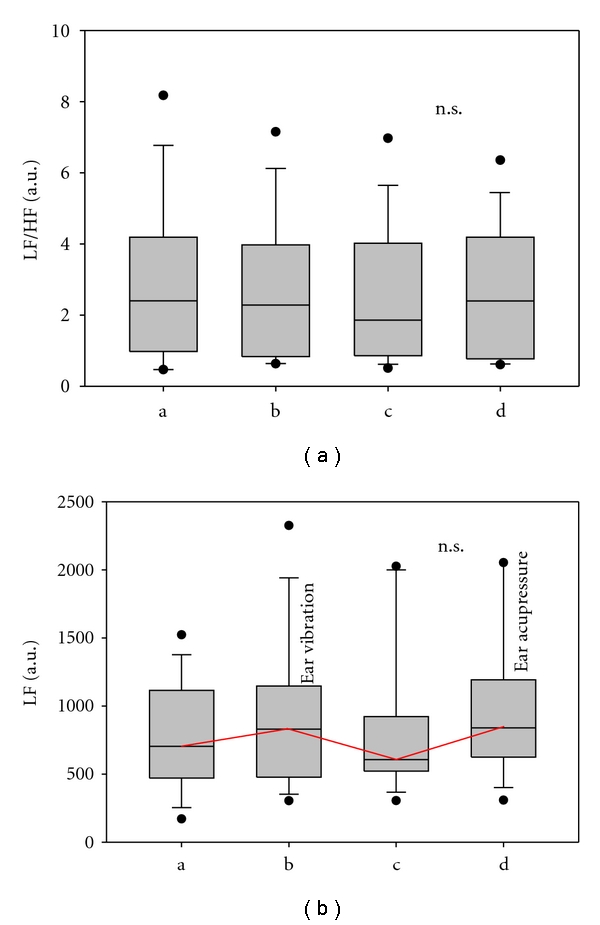
(a) LF (low frequency)/HF (high frequency) ratio. (b) the LF (low frequency) band of HRV. Note that the median of the LF parameter increases during ear acupressure vibration and during manual ear acupressure in 14 subjects. For further explanation, see Figures 3 and 4.

Furthermore, the biosignal monitoring during acupressure (vibration) and manual acupressure showed substantial increases in the LF frequency band (Figure 5(b)). The LF/HF ratio alterations were insignificant (Figure 5(a)).

**Figure 6:**
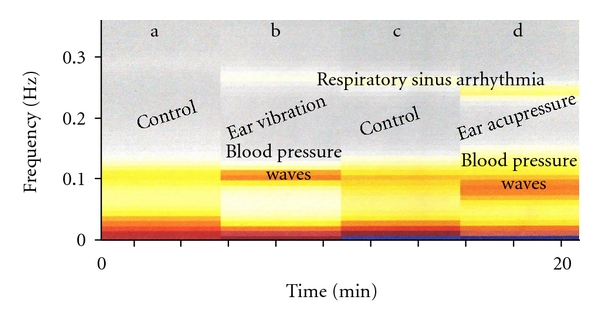
Frequency analysis of heart rate variability. Note the appearance of the influence of blood pressure modulation (*∼*0.1 Hz) during “b” (ear acupressure vibration) and “c” (manual acupressure). For further explanation, see Figure 3.

**Figure 7:**
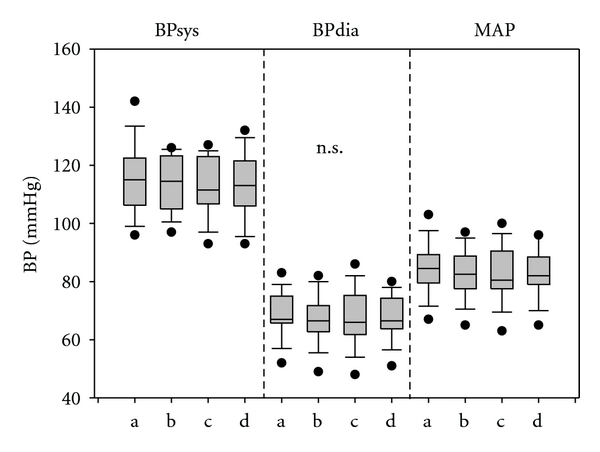
Systolic blood pressure (BPsys), diastolic blood pressure (BPdia), and mean arterial pressure (MAP) of the 14 healthy volunteers during the different phases (a–d). For further explanation of the box plots, see Figure 4.

A typical example from the new software analysis is shown in Figure 6. In this person (27-year-old male subject), strong influences of blood pressure waves (*∼*0.1 Hz) appear in the frequency analysis of the HRV during and after the stimulation phases. Additionally, the influence of the respiratory sinus arrhythmia is demonstrated (*∼*0.27 Hz). The analysis of the blood pressure is shown in Figure 7. No significant changes caused by stimulation were found.

**Figure 8:**
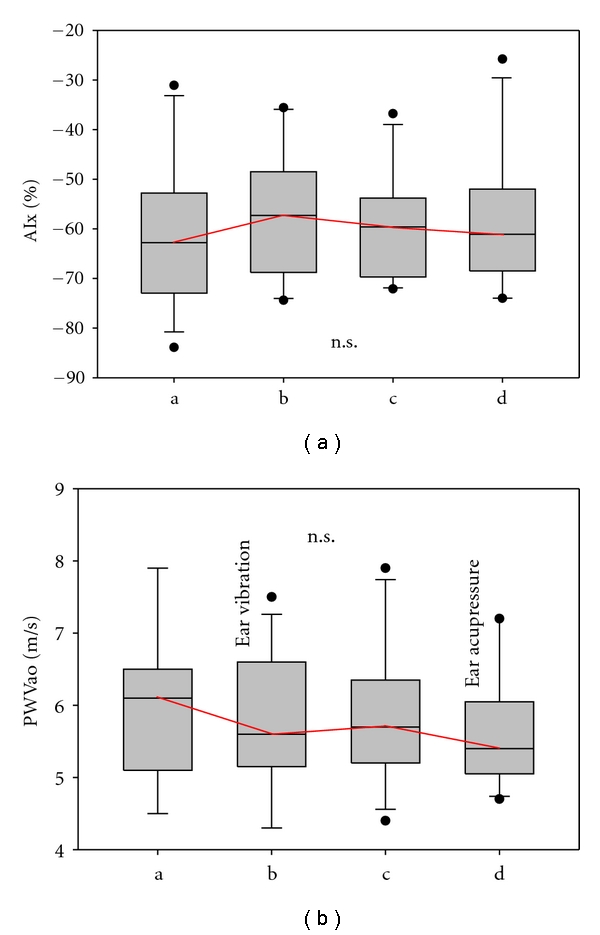
(a) brachial augmentation index (AIx) in %, describing the influence of the reflected pulse wave on systolic pressure (in percent of pulse pressure) of the 14 healthy volunteers during measurement phases a–d (see Figure 3). (b) PWVao (aortic pulse wave velocity in m/s) in the same subjects. For further explanation of the box plots, see Figure 4.

Figure 8(a) summarises the preliminary results of the parameter AIx for the 14 participants in this pilot study. The AIx values increased during acupuncture vibration; however, statistical significance was not reached. The velocity of the pulse wave between the aortic root and the bifurcation of the aorta in m/s is demonstrated in the same Figure 8(b). There was a continuous insignificant decrease in PWVao (aortic pulse wave velocity) during the recording procedure.

## 4. Discussion

Auricular acupuncture is used for various autonomic disorders in clinical practice in western and eastern medicine. Recently, there is a growing focus on the important role of the brain, and, therefore, there is also a need to explain how acupuncture and acupuncture-like stimulations affect the cerebral autonomic function. There is strong evidence from previous animal and human studies that acupuncture impacts the autonomic nervous system. There are two important publications from Gao et al. [17, 18] describing experiments in animal models. The first study [17] aims to examine the effects of acupuncture stimulation at different auricular areas on cardiovascular and gastric responses. In male anesthetised Sprague-Dawley rats, stimulation with manual acupuncture was performed. The authors found that the biggest depressor response was evoked from an area that corresponds to the “heart” stimulation point in humans that was used in our present investigation. The results from Gao et al. [17] also show that similar patterns of cardiovascular and gastric responses could be evoked by stimulation of different areas of the auricle. Their results do not support the theory of a highly specific functional map in the ear. Rather, there is a similar pattern of autonomic changes in response to auricular acupuncture with variable intensity depending on the area of stimulation [17]. Due to these previous results, we used two different active stimulation methods applied at the same acupoint, and we did not perform acupressure at a control point localised closed to the stimulation area.

The second study from Gao et al. was published recently in 2011 in Brain Research [18] and, as already mentioned above, showed that auricular acupuncture induces cardiovascular inhibition, increases the response of cardiac-related neurons in the nucleus tractus solitaries, and evokes cardiovascular inhibition through the baroreceptor reflex mechanism. Acupuncture-like stimulation was repeated in 58 male Sprague-Dawley rats in the area of the “heart” auricular point. In contrast to our investigation in humans, the authors of this study recorded invasive arterial pressure and HR to detect the cardiovascular response induced by auricular acupuncture. They could clearly show that acupuncture at the “heart” auricular point regulates cardiovascular function by activating the cardiac-related and depressor neurons in the nucleus tractus solitaries in a manner similar to the baroreceptor reflex in cardiovascular inhibition [18].

Experimental studies concerning ear acupuncture-like stimulation at the “heart” acupoint in humans are rare. In a Chinese study, the authors investigated 30 patients with hypertension. A comparison of the hypotensive short-term effects between the “heart” ear point and another point of ear needling showed that there was a marked hypotensive effect associated with stimulation of the “heart” point [15].

In 1993, Zhou [16] investigated the effect of auriculo-acupuncture plus needle embedding in the “heart” point on left cardiac, humoral, and endocrine function. Twelve patients with heart failure complicated by dilating cardiomyopathy were divided randomly into an auriculoacupuncture group (*n* = 7) and controls (*n* = 5). Left cardiac function and plasma levels were measured. The results of that study indicated that auriculoacupuncture plus needle-embedding in the “heart” acupoint could improve the left cardiac function in patients with heart failure complicated by dilating cardiomyopathy and that the function of an acupoint is distinctly different from that of a nonacupoint [16].

Concerning our present study, it is important to point out that the RR-intervals in the ECG are controlled by the blood pressure control system, which is influenced by the hypothalamus and, in particular, by the vagal cardiovascular centre in the lower brainstem [6, 14]. Calculation of the ECG power spectra is thought to provide an understanding of the effects of the sympathetic and parasympathetic systems on HRV. Some of the frequency bands in the spectrum of the HRV could be interpreted as markers of physiological relevance. Several of the associated mechanisms are thermoregulatory, which can be found in the very low frequency band, blood pressure and respiratory effects [6, 14]. The following influences can be distinguished for different ranges of HRV: (a) respiratory sinus arrhythmia (approx. 0.15–0.5 Hz), including central nervous system respiratory impulses and interactions with pulmonary afferents; (b) the so-called “10-s-rhythm” (approx. 0.05–0.15 Hz), which describes the natural rhythm of active cardiovascular neurons in the lower brainstem (the circulatory centre and its modulation by feedback with natural vasomotor rhythms via baroreceptor feedback); (c) longer wave HRV-rhythms (approx. <0.05 Hz), such as effects from the renin angiotensin system and temperature regulation as well as metabolic processes [4, 14].

In the present study, HR decreased significantly and HRV total increased during both ear acupressure and ear vibration (compare Figure 4(b)). Manual acupressure had more of an effect on HRV than the application of vibration stimuli. The analysis of the LF frequency band also showed a marked increase during stimulation. This increase is mainly based on the intensification of the related mechanism of the blood pressure regulation (10-s-rhythm). Figure 6 clearly demonstrates the appearance of this influence. 

The present study also includes a new application of the innovative oscillometric technique for measuring arterial stiffness in the field of acupuncture. As of May 2011, only six scientific articles concerning “arterial stiffness and acupuncture,” “wave reflection and acupuncture,” and “pulse wave velocity and acupuncture” could be found in the scientific literature [6, 19–23]. 

Scientists from Austria, China, Japan, Mexico, and Taiwan (alphabetical order) performed these studies. 

The PWV and AIx increase in somewhat different ways in parallel with the aging process, and they provide different information regarding the arterial vascular status [23, 24]. Both parameters provide extensive information on the arterial vascular system, and the prognostic significance of arterial stiffness is expected to be high [5]. The results from our pilot study conducted in 14 healthy volunteers regarding the acute effects of auricular acupressure on human arterial stiffness and wave reflection showed a minor and insignificant increase in the brachial AIx after acupressure vibration (see Figure 8(a)) and a decrease in the aortic PWV immediately after acupressure vibration or manual acupressure stimulation (see Figure 8(b)). 

However, there are some limitations of this pilot study. The number of subjects was small (*n* = 14), and there was no control group with a control acupuncture point. As mentioned at the beginning of the discussion section, previous results from a study by Gao et al. [17] showed that it is difficult to identify an ear placebo point for such investigations Therefore, based on the results of this pilot study and of other previous studies [6, 19–23], we intend to conduct a larger study to confirm or refute these preliminary findings. Our hypothesis is that ear acupressure (manual acupressure or acupressure vibration stimulation) can influence the autonomic nervous system. We believe that it is possible that these ear stimulation methods may cause measurable, reproducible physiological alterations, especially of HR, HRV, and blood pressure, as well as changes in the parameters of human arterial stiffness and wave reflection. These latter responses have only been used in two studies [6, 19] on needle body acupuncture. The present study is the first investigation of noninvasive parameters in humans using ear acupuncture-like stimulation. Therefore, further investigations are necessary. With reference to the present study, differences between the effects of needle acupuncture and acupressure on the parameters mentioned above also a matter of future research.

Ear acupuncture has been used for medical treatment for thousands of years. A large amount of empirical data is available, but the quantification of the effects on the brain and the periphery has not previously been possible. Using modern biomedical techniques, changes in vital parameters can now be quantified in a noninvasive way. Modernisation of acupuncture at the Medical University of Graz [1–4, 6, 25–39] has been achieved, and research on this topic is underway.

## 5. Conclusion

The following conclusion can be drawn from the results of this study: HR decreases and HRV total increases significantly during ear acupressure and/or ear acupressure vibration. The velocity of the pulse wave between the aortic root and the bifurcation of the aorta decreases markedly (yet insignificantly), whereas the augmentation index increases immediately after acupressure vibration and manual acupressure at the “heart” auricular acupoint. Our hypothesis as stated in the Discussion will require future investigation for verification.
